# Biotization of *in vitro* oil palm (*Elaeis guineensis* Jacq.) and its plant-microbe interactions

**DOI:** 10.3389/fpls.2023.1150309

**Published:** 2023-04-18

**Authors:** Shey-Li Lim, Sreeramanan Subramaniam, Md Abdul Baset Mia, Abdul Rahman Siti Rahmah, Amir Hamzah Ahmad Ghazali

**Affiliations:** ^1^ School of Biological Sciences, Universiti Sains Malaysia, Minden, Penang, Malaysia; ^2^ Department of Crop Botany, Faculty of Agriculture, Bangabandhu Sheikh Mujibur Rahman Agricultural University, Gazipur, Bangladesh; ^3^ Advanced Biotechnology and Breeding Centre, Malaysian Palm Oil Board, Persiaran Institusi, Bandar Baru Bangi, Kajang, Selangor, Malaysia

**Keywords:** biotization, micropropagation process, plant growth promoting rhizobacteria, *in vitro* plant development, biotic and abiotic stress

## Abstract

Continuous discovery of novel *in vitro* plant culture practices is always essential to promote better plant growth in the shortest possible cultivation period. An alternative approach to conventional micropropagation practice could be achieved through biotization by inoculating selected Plant Growth Promoting Rhizobacteria (PGPR) into the plant tissue culture materials (e.g., callus, embryogenic callus, and plantlets). Such biotization process often allows the selected PGPR to form a sustaining population with various stages of *in vitro* plant tissues. During the biotization process, plant tissue culture material imposes developmental and metabolic changes and enhances its tolerance to abiotic and biotic stresses, thereby reducing mortality in the acclimatization and pre-nursery stages. Understanding the mechanisms is, therefore crucial for gaining insights into *in vitro* plant-microbe interactions. Studies of biochemical activities and compound identifications are always essential to evaluate *in vitro* plant-microbe interactions. Given the importance of biotization in promoting *in vitro* plant material growth, this review aims to provide a brief overview of the *in vitro* oil palm plant-microbe symbiosis system.

## Introduction

**Figure 1:**
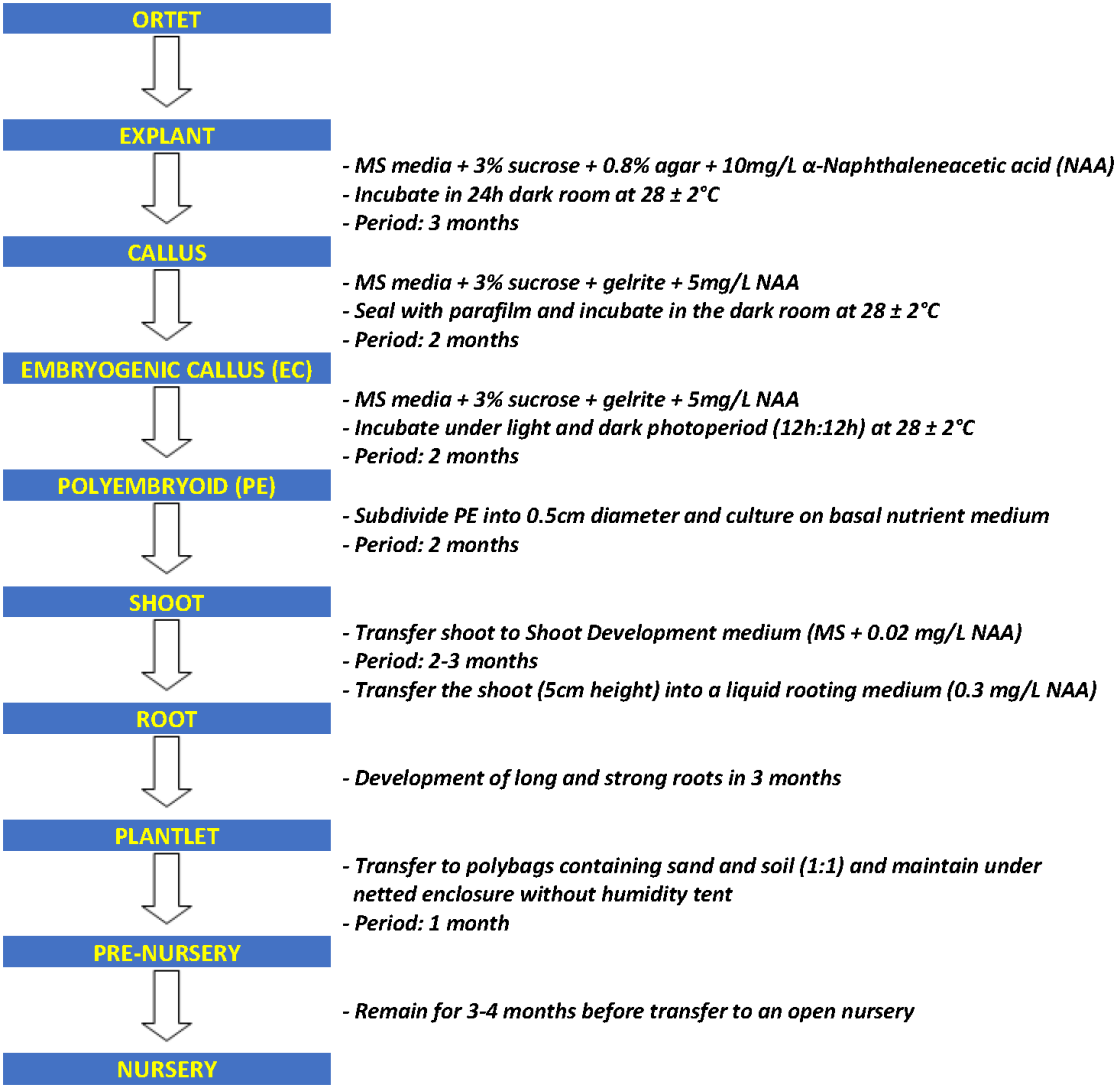
Flow chart of oil palm tissue culture process.

The cultivation of oil palm (*Elaeis guineensis* Jacq.) has highly expanded in recent years, and it is now the major source (~40%) of the world’s vegetable oil supply (Meijaard et al., 2020). Crude palm oil production in Malaysia increased from 4.1 million tonnes in 1985 to 16.9 million tonnes in 2010 and reached 19.14 million tonnes in 2020 (Malaysian Palm Oil Board, 2010; Parveez et al., 2022). To date, the industry contributes 8% to the country’s gross national income. Despite its remarkable contribution to the economy, there has not been much progress in yield improvement in the last four decades as the CPO in a recent production of 3.16 t/ha/year in 2021 (Parveez et al., 2022). With the current planted area of 5.74 million hectares and mature palms covering 89.7% (Parveez et al., 2022), close to reaching the targeted limit of 6.5 million hectares introduced by the Malaysian government in committing to sustainability (Wan Mohd Jaafar et al., 2020), extensive efforts are still being carried out to sustain and improve the higher productivity of the industry. Therefore, with limited land for replanting, increasing yield per unit of the land area is inevitable. Among the efforts that have been carried out include the opening of new oil palm plantations and the planting of high-quality oil palm plantlets produced through tissue culture techniques. Through breeding, annual yield increases at an average of 1.5% (Woittiez et al., 2017), while cloning high-yielding palms boasts a potential yield increase of 10–15% (Soh, 2018). Thus, the demand for *in vitro* oil palm planting materials has increased by 20–30% compared to seedling planting materials (Soh et al., 2001). However, the existing micropropagation process still has certain restrictions due to the limitation of the selection of explant materials and the low rate of differentiation and regeneration of somatic embryogenesis (Weckx et al., 2019). It is also a time-consuming process with all of the oil palm tissue culture steps required to generate plantlets from explants taking nearly a year to complete (**Figure 1**) (Rohani et al., 2000), together with the inefficiency of callus and embryogenesis development. Therefore, there is an urgent need to discover a new technique to increase the yield of oil palm tissue culture material.

Introduction of naturally existing endophytic bacteria into plant tissue culture materials could improve plant cell proliferation and differentiation. Increased plant biomass due to bacterial inoculation have been observed in various plant species (e.g., strawberry, wheat, rice, sorghum, tomato, soybean, maize, etc.) (Pérez-Montaño et al., 2014; Mitter et al., 2013). For decades, Plant Growth Promoting Rhizobacteria (PGPR) such as *Acetobacter diazotrophicus* R12, *Azospirillum brasilense* Sp7 (ATCC 29729), *Herbaspirillum seropedicae* Z78 (ATCC 35893), *Bacillus sphaericus* (UPMB10), and *Azospirillum lipoferum* (CCM3863) have been widely reported and used as the biofertilizers in the oil palm plantlets to promote plant growth (**Table 1**) (Amir et al., 2001; Azlin et al., 2007; Lim et al., 2010; Noor et al., 2013; Lim et al., 2018b). Through these findings, all the microbial inoculants increased the growth and nutritional assimilation (total N) of oil palm plantlets and at the same time also improved soil properties. Not only in oil palm plantlets, studies also showed that *H. seropedicae* can fix the significant amount of N_2_ and contribute to plant growth in sugarcane, rice, and oil palm (Boddey et al., 1995; Elbeltagy et al., 2001; Gyaneshwar et al., 2002; James et al., 2002; Roncato-Maccari et al., 2003; Ai’shah et al., 2009).

**Table 1:** Summary of biotization in tissue cultured oil palm materials with plant growth promoting rhizobacteria (PGPR).

Oil palm variant	Type of plant culture	Bacterial species	Inoculum	Effect of interaction	Reference
*Elaeis guineensis* var. *tenera*	Plantlet	*Acetobacter diazotrophicus* R12	Broth culture	• Increment of total fresh weight (19.50%) • Higher acetylene reduction assay and leaf chlorophyll content compared to the N free MS medium	(Azlin et al., 2007)
	Plantlet	*Azospirillum brasilense* Sp7	Broth culture	• Increment of total dry weight (12.20%) • Increment of root volume (82.35%) • Higher quantum yield and net photosynthesis rate compared to the control (killed inoculum Sp7)	(Amir et al., 2001)
				• Increment of total fresh weight (49.81%) • Higher acetylene reduction assay and leaf chlorophyll content compared to the N free MS medium	(Azlin et al., 2007)
	Plantlet	*Azospirillum lipoferum* (CCM3863)	Broth culture	• Increment of total dry weight (34.63%) • Increment of root volume (41.17%) • Caused a significant increase (16%) in shoot nitrogen concentration compared to the control (killed inoculum Sp7)	(Amir et al., 2001)
	Plantlet	*Microbacterium* sp. E7	Broth culture	• Increment of shoot fresh weight (23.40%), shoot height (51.46%), and shoot protein content (90.29%) compared to the control (killed inoculum Z78)	(Noor et al., 2013)
	Plantlet	*Acetobacter* sp. E9	Broth culture	• Increment of shoot fresh weight (23.40%), shoot height (17.84%), and shoot protein content (27.18%) compared to the control (killed inoculum Z78)	(Noor et al., 2013)
	Plantlet	*Microbacterium* sp. E14	Broth culture	• Increment of shoot fresh weight (28.94%), shoot height (35.38%), and shoot protein content (121.36%) compared to the control (killed inoculum Z78)	(Noor et al., 2013)
	Plantlet	*Herbaspirillum seropedicae* Z78	Broth culture	• Increment of shoot fresh weight (55.32%), shoot height (52.8%), and shoot protein content (129.13%) compared to the control (killed inoculum Z78)	(Noor et al., 2013)
	Embryogenic callus	*Herbaspirillum seropedicae* Z78 (ATCC 35893)	Pellet cell	• Decreased biomass fresh weight (36.74%) • All embryoids were yellowish brown compared to the control (full N)	(Lim et al., 2016a)
			Supernatant	• Increment of biomass fresh weight (45.19%) • Embryoid formation was opaque white (21.23%) and yellowish brown (78.77%) compared to the control (full N)	
			Broth culture	• Increment of biomass fresh weight (39.22%) • Embryoid formation was opaque white (29.60%) and yellowish brown (70.40%) compared to the control (full N)	
			Sonicated cell	• Increment of biomass fresh weight (64.89%) • Embryoid formation was opaque white (35.40%) and yellowish brown (64.60%) compared to the control (full N)	
*Elaeis guineensis* var. *dura*	Callus, Embryogenic callus	*Herbaspirillum seropedicae* Z78 (ATCC 35893)	Sonicated cell	• Increment of callus fresh weight (11.21% and 26.76%) compared to the control (limited N) • Increment of embrygenic callus fresh weight (61.07% and 60.33%) compared to the control (limited N)	(Lim et al., 2016b; Lim et al., 2018a)
			Pellet cell	• Increment of callus fresh weight (114.10% and 147.87%) compared to the control (limited N) • Decreased of embrygenic callus fresh weight (15.26% and 32.26%) compared to the control (limited N)	
	Plantlet		Sonicated cell	• Increment of total biomass dry weight (42.18%) compared to the control (limited N)	(Lim et al., 2018b)
			Broth culture	• Increment of total biomass dry weight (68.31%) • Higher total protein content • Higher IAA concentration in root • Increment in root volumn compared to the control (limited N)	
			Pellet cell	• Increment of total biomass dry weight (55.44%) • Higher IAA concentration in leaves compared to the control (limited N)	
*Elaeis guineensis* (Deli x Yangambi)	Plantlet	*Bacillus sphaericus* (UPMB10)	Broth culture	Functional classification of tentative unique genes that were upregulated. • Protein synthesis and processing (28%) • Stress-related protein (22%) • Primary metabolism (13%) • Cell division (7%) • Membrane transport (4%) • Signal transduction (4%) • Gene expression and RNA metabolism (2%) • Vesicular trafficking, protein sorting, and secretion (2%)	(Lim et al., 2010)

However, to date, less attention has been paid to the biotization processes of other *in vitro* culture materials such as callus and embryogenic callus. Not long ago, the potential of the PGPR to promote *in vitro* proliferation and differentiation of plant culture materials such as callus and embryogenic callus was reported in oil palm and wheat (Lim et al., 2016a; Lim et al., 2016b; Tkachenko et al., 2021). Selected beneficial bacteria can accelerate the growth and development of *in vitro* plant culture *via* a highly sustainable and environmentally friendly approach. Thus, biotization process involving PGPR is highly prospective to increase the effectiveness of the micropropagation process. This novel technique for *in vitro* oil palm culture involving PGPR can improve the efficiency of the micropropagation process and may provide a solution to the ineffective callus and embryogenesis production. Due to this consideration, this review will mainly focus on how plant-microbe interactions improve oil palm micropropagation.

## Colonization of PGPR for *in vitro* oil palm plant materials

A vital feature of all PGPRs is the creation of a symbiotic environment that is effective for colonization. However, the success of invasion and colonization in the host also requires bacteria to overcome plant defense responses triggered by microbial recognition. To further understand the symbiosis system, the adhesion of PGPR in plant tissue culture materials will be discussed here. Upon biotization, PGPRs tend to colonize the intercellular spaces of the co-cultured *in vitro* plant materials, forming a plant-microbe symbiosis system. This is a key step to establishing an artificial symbiotic relationship between *in vitro* plant material and PGPR. Studies by Lim et al. (2016a) and Lim et al. (2016b) proved that the cells of *H. seropedicae* Z78 colonized the surface and the intercellular spaces of the treated callus and embryogenic callus of oil palm. The high concentration of carbon sources in the apoplast may contribute to the successful colonization of bacteria in the intercellular spaces of plant cells (Bennett and Lynch, 1981; Schmidt et al., 2011). To create a successful symbiotic relationship, plant cells need to provide bacteria with carbon and energy sources by converting available sugar in the culture medium during the biotization process (Preininger et al., 1997).

Biotization process could also enhance the development of the root systems of the *in vitro* plantlet. A fully functional root system is also vital for acquiring minerals, nutrients, and water and responding to stress signals under micropropagated conditions and during the acclimatization stage (Maksimov et al., 2011). As reported by Azlin et al. (2007), *A. diazotrophicus* R12 and *A. brasilense* Sp7 were observed to colonize the root of the epidermis cell of oil palm plantlets. Colonized roots showed higher fresh weight and nitrogenase activity, which suggests the efficiency of PGPR in fixing atmospheric nitrogen. Amir et al. (2001) and Lim et al. (2018b) also reported a significant increase in root volume for oil palm plantlets treated with *A*. *brasilense* Sp7*, A. lipoferum* CCM3863, and *H. seropedicae* Z78 broth culture, respectively, suggesting that the PGPRs stimulate root growth and development.

To successfully colonize the host plant, lipopolysaccharide (LPS), a component of endophytes plays a key role in plant colonization and forms artificial plant-microbe interactions (Serrato et al., 2010; Krasova et al., 2022). Other bacterial cell components such as flagella proteins, exopolysaccharides and adhesins are crucial in the interaction between the PGPR with higher plants. Such components are the first structures that come into contact with the host plant (Balsanelli et al., 2013). Balsanelli et al. (2016) reported that *H. seropedicae* interacted with the host plant through a specific chemotaxis system, possibly attaching to the root surface with type IV pili and the lipopolysaccharide. After completion of the root surface colonization, potential PGPR could colonize roots internally *via* lateral root emergence sites and root tips and meristems (Botta et al., 2013). However, studies on PGPR colonization of *in vitro* plant material, especially in callus and embryogenic cells, are very limited. Detailed studies of PGPR colonization with *in vitro* oil palm culture material appear to be eagerly needed to provide a better understanding of the interaction.

## Artificial symbiosis of PGPR and *in vitro* plant culture

After colonization, it has been proven that selected PGPR could established an artificial symbiotic relationship with *in vitro* plant culture. This relationship improves the micropropagation process by changing the physiology of the plant material and increasing the yield of produced secondary metabolites. Inoculated bacteria can eventually increase the availability of nutrients and phytohormones and reduce any potential biotic and abiotic plant stresses faced by plant tissue culture material. An artificial symbiosis between selected diazotrophic PGPR and *in vitro* plant materials is a viable solution for transferring the inoculum to newly selected hosts (Varga et al., 1994). If the cultural conditions are refined, thus, a successful symbiotic relationship between plant cells and PGPR can be created (Maksimov et al., 2011). Sturz et al. (2000) suggested that such a relationship could be introduced to the *in vitro* plant materials as early as the embryoid and shoot formation stages. At an early stage, this relationship allows the host plant to be better adapted to the inoculum for an effective and efficient micropropagation process (Sturz et al., 2000; Azlin et al., 2007).

Some early micropropagation stages of plant-microbe interactions have also been reported, such as *Rhizobium japonicum* associated with tobacco and cowpea cell cultures (Scowcroft and Gibson, 1975), *Azotobacter zettuovii* and carrot callus (Varga et al., 1994), *Azomonas insignis* with strawberry callus (Preininger et al., 1997), *Azospirillum* sp. and *Gluconacetobacter* sp. in sugarcane callus (Anand Kumar et al., 2004), rhizobacteria with saffron cormlet (Parray et al., 2015), *H. seropedicae* Z78 with oil palm callus and embryogenic callus (var. *tenera* and *dura*) (Lim et al., 2016a; Lim et al., 2016b), and *A*. *brasilense* with wheat callus (Tkachenko et al., 2021). All these studies reported that the selected PGPRs were found to be able to promote plant growth and further improve the efficiency of the micropropagation process by enhancing the cell division and proliferation. In contrast, several reports revealed that biotization of *in vitro* plant material can have detrimental effects to plant growth. According to Krasova et al. (2022), viable *A*. *brasilense* Sp7 culture challenged wheat callus resulted in cell death after 7 days of cultivation. Low biomass was also observed in oil palm embryogenic callus (var. *tenera*) treated with *H. seropedicae* Z78 pellet cells (Lim et al., 2016a). This suggests that not all forms of PGPR are suitable for establishing bioization with certain plant species or plant varieties. Despite the makeup of biotization may vary depending on the tissue and developmental stage of the plant itself, the composition of the endophytic population is always not necessarily plant species-specific. However, screening and selection are always required to ensure the PGPR is appropriate for certain species. From a conceptual point of view, the most ideal approach would be using the locally putative endophytes which isolated from the same plant species or varieties. All these microbes occupying the endospheric of plants are most likely to be selected as niches by the plant cells themselves. This is because these microbes appear to be able to form relationships with host plants and circumvent the effects of plant defense metabolites (Senthilkumar et al., 2011).

## PGPR promotes somatic embryogenesis

Selected PGPRs can synthesize phytohormones which are capable of affecting the hormonal balance of plants. Interestingly, the interaction of plants and bacteria enables the production of phytohormones and compounds mimicking the natural plant growth regulator (Brader et al., 2014). Auxin such as indole-3-acetic acid (IAA) is mainly considered to be the most critical phytohormone in plant tissue culture which affects cell division, elongation, differentiation, and the initiation of organ formation of plant tissues (Spaepen et al., 2007). IAA produced by *Herbaspirillum* sp. is one of the mechanistic pathways that could contribute to plant growth promotion (Bastián et al., 1998). Bacterial IAA loosens plant cell walls and increases root secretions, thereby providing additional nutrients to support bacterial growth (James et al., 2002; Chi et al., 2005). It has been suggested that the PGPR could overcome abiotic stresses by providing them with IAA that directly stimulates plant growth (Glick, 2012). Lim et al. (2018b) revealed that auxin activation may be a mode of cellular adaptation, which leads to the embryogenic capacity of somatic embryogenesis, and demonstrated the importance of a co-culture system of PGPR and oil palm tissue culture material. All these findings significantly demonstrate the ability of selected inoculum to exert high levels of auxin when associated with plant tissue and promote callus differentiation and embryogenic callus formation in the somatic embryogenesis process of oil palm. In the cross-section of oil palm callus treated with *H. seropedicae* Z78 cells, isodiametric cells formed meristematic fragmented segments composed of compact tissues surrounded by densely stained cytoplasm and nucleus. This indicates that intense cellular division occurred in the differentiated region of the treated callus (Lim et al., 2016b). Under such conditions, meristems can further develop into embryogenic clumps, as small and isometric cells with dense cytoplasm and starch accumulation indicate progression toward embryogenesis (Pádua et al., 2013; de Carvalho Silva et al., 2014). This demonstrates that inoculated *H. seropedicae* Z78 pellet cells provide IAA to the oil palm callus tissues and eventually promote cell division.

Inoculation of microorganisms into the culture can also create stress for the plant tissue (Karami and Saidi, 2010). Stress is one of the factors that promote embryogenic cell differentiation. Lim et al. (2018a) reported that the detection of phenolic compounds in inoculated oil palm callus and embryogenic callus could be indicated as one of the stress factors for plant cells. The phenolic compounds benefit plant cells as modulators for IAA synthesis, where phenolic compounds inhibit the enzymatic oxidation of IAA and results in plant cell division and differentiation (López Arnaldos et al., 2001). According to Zavattieri et al. (2010), the parallel induction of these co-cultured stresses can lead to the development of morphological changes during the transition of somatic embryogenesis in rapid differentiation. This highlighted that the interaction between auxin and stress signalling, which results in somatic embryogenesis formation, could reprogram behaviour at different levels. However, the mechanism by which these stresses stimulate the differentiation of embryogenic cells is still not much reported.

Diazotrophs can provide fixed nitrogen required by the PGPR associated host plant (Mitter et al., 2013). Nitrogen fixing bacteria like *H. seropedicae* Z78 have been identified and isolated from the rhizosphere of oil palm. Baldani and Baldani (2005) and Chubatsu et al. (2012) also reported the ability of *H. seropedicae* able to fix N_2_ and to promote plant growth through phytohormone excretion in an associative condition. This native putatively PGPR was also reported to have the ability to carry out nitrogenase activity when associated with oil palm callus, embryogenic callus, and plantlets (Lim et al., 2016b; Lim et al., 2018b). This suggests that *H. seropedicae* Z78 is a potential PGPR that accelerates *in vitro* oil palm material. Overall, microbial co-cultivation for *in vitro* plant culture may impose developmental and metabolic changes on macrosymbionts and enhance their tolerance to abiotic and biotic stresses.

## Biochemical activities of inoculated *in vitro* oil palm

To promote plant growth, one of the important compounds is amino acid, which is essential in protein synthesis (Vidal and Gutierrez, 2008). Lim et al. (2018a) showed an increase in total amino acid content and biomass at day 30 after treatment of callus with *H. seropedicae* Z78 pellet cells, suggesting an accelerated rate of formation of friable embryogenic callus. The increase in total free amino acids in tissue may be due to increased metabolic activities in plant cultures, leading to cell division and increased cellular biomass in somatic embryogenesis (Feher et al., 2003). Aberlenc-Bertossi et al. (2008) and Sghaier-Hammami et al. (2009) also showed that during embryo maturation, proteins of the glycolytic pathway, as well as storage proteins start to be expressed in date palm and oil palm, respectively. These reports suggested that an active protein synthesis step may take place in the embryogenic callus, which might be essential for the differentiation of somatic embryos.

Total sugar content is another important feature and is a carbon source for polysaccharides synthesis. According to Drogue et al. (2012), sugars in specific bacterial lipopolysaccharide are involved in the colonization and attachment of plant microbes. Baud et al. (2002) showed that sugar mobilization is very important during the early stage of embryogenic development, as the sugars serve as the main sources of carbon skeleton and metabolic signalling. Gomes et al. (2014) and Warchoł et al. (2015) also observed an increase in total soluble sugar levels at the somatic embryo maturation stage of the oil palm and cabbage tree. Regulation of plant cell morphology at transcriptional, translational, and post-translational levels through cell differentiation depends on the sensitivity of plant tissues to transport at different concentrations and levels of sugar (Delrot et al., 2000). However, it is difficult to differentiate between the metabolic function and regulation of sugars in plant cells, and indirect alterations in the sugar metabolism that may also affect plant cell morphogenesis (Lozovaya et al., 2006). Despite the potential and current understanding of PGPRs and *in vitro* plants, existing research data cannot fully explain the interaction mechanism at the biochemical level. Any changes in the host plant’s biochemical profile could further explain the function of PGPR in the interaction.

## Conclusions and future perspectives

**Figure 2:**
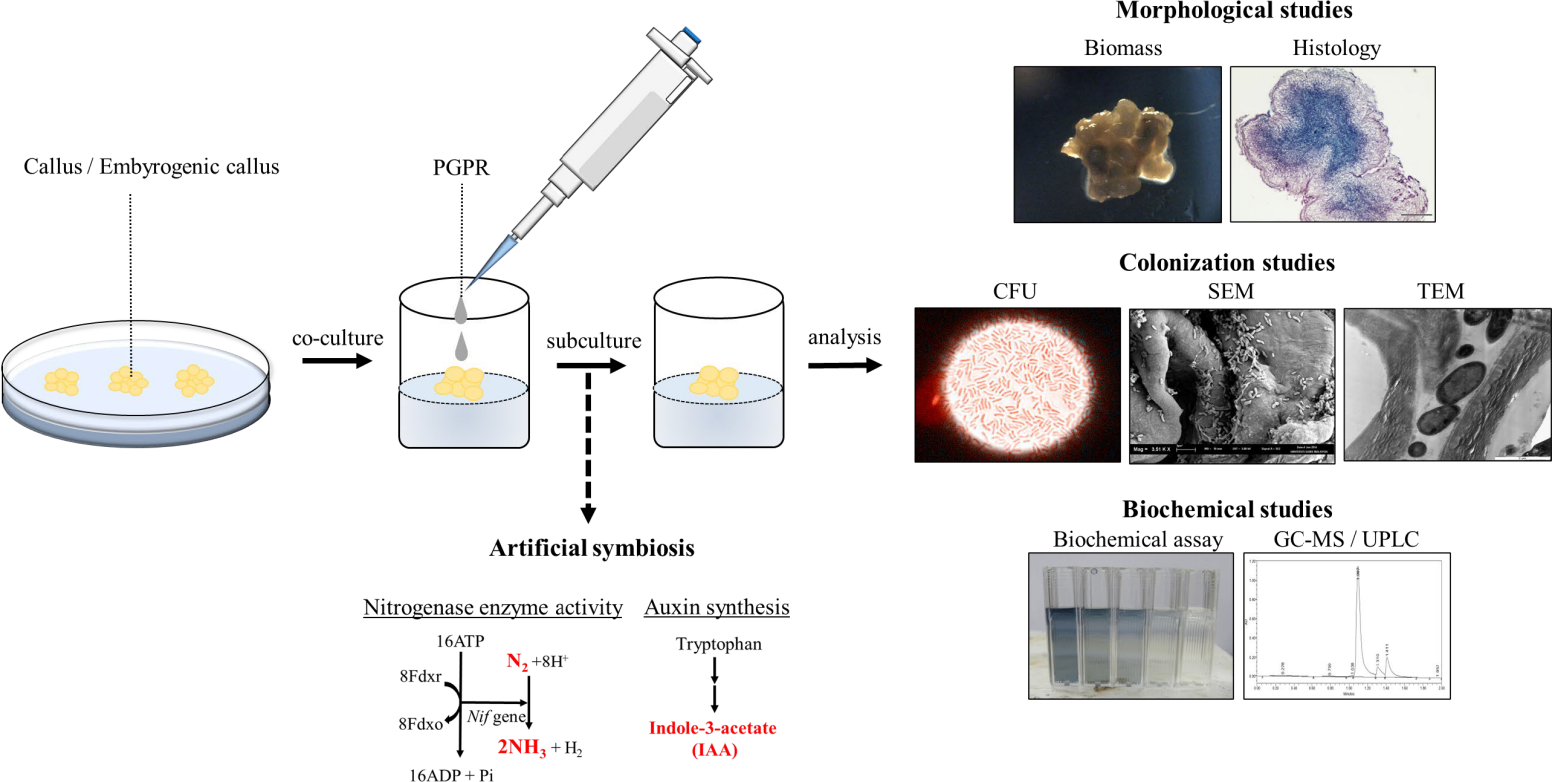
Schematic diagram of the biotization process. Artificial symbiosis can be created *via* co-culture of selected PGPR with *in vitro* plant materials. Morphological studies, colonization studies, and biochemical studies are necessary assays to identify the *in vitro* plant material-microbe interactions. PGPR, plant growth promoting rhizobacteria; CFU, colony form unit; SEM, scanning electron microscope; TEM, transmission electron microscope; GC-MS, Gas chromatography-mass spectrometry; UPLC, ultra-performance liquid chromatography.

Over the years, the optimization of tissue culture technology in oil palm has reached a bottleneck, the long oil palm tissue culture process (**Figure 1**) and low yield of callus and embryogenic callus remain to limit the production of high-quality plantlets. For decades, PGPR has been reported as an effective microbial inoculant capable of promoting plant growth. Owing to their natural properties, PGPRs appear to be a good choice for accelerating or at least improving plant tissue culture production. A schematic diagram is provided in this review to describe the flow of inoculation and experimental procedures (**Figure 2**). The preliminary step in obtaining an ideal match of PGPR to the host plant is to screen PGPR of its biochemical ability. Furthermore, the PGPR colonization process is rather important for establishing a mutual plant-microbe interaction. Rhizobacterial quorum sensing appears to play a role in the colonization process. It would be an interesting extension topic if PGPRs could somehow generate the quorum-sensing signal in association with the host plants, and further promote plant cell development. Understanding the relationship between *in vitro* plant-microbe interactions at the molecular level is critical. Studies such as proteomics, RNA seq, or microarrays can be performed to further identify potential proteins and enzymes involved in the interactions. In brief, extended studies are always needed to discover more potential PGPRs and create a stable symbiotic environment between oil palm tissue culture and PGPRs.

## Author contributions

AG and S-LL conceived the idea. AG, S-LL, SS, BM and ARSR wrote and revised the manuscript. All authors contributed to the article and approved the submitted version.
